# A High Resolution Radiation Hybrid Map of Wheat Chromosome 4A

**DOI:** 10.3389/fpls.2016.02063

**Published:** 2017-01-10

**Authors:** Barbora Balcárková, Zeev Frenkel, Monika Škopová, Michael Abrouk, Ajay Kumar, Shiaoman Chao, Shahryar F. Kianian, Eduard Akhunov, Abraham B. Korol, Jaroslav Doležel, Miroslav Valárik

**Affiliations:** ^1^Institute of Experimental Botany, Centre of the Region Haná for Biotechnological and Agricultural ResearchOlomouc, Czechia; ^2^Institute of Evolution, University of HaifaHaifa, Israel; ^3^Department of Plant Sciences, North Dakota State University, FargoND, USA; ^4^Biosciences Research Laboratory, United States Department of Agriculture-Agricultural Research Service, FargoND, USA; ^5^Cereal Disease Laboratory, United States Department of Agriculture-Agricultural Research Service, University of Minnesota, St. PaulMN, USA; ^6^Department of Plant Pathology, Kansas State University, ManhattanKS, USA

**Keywords:** endosperm radiation hybrid panel, radiation hybrid map, wheat chromosome 4A, chromosome deletion bin map, *Triticum aestivum*, SNP iSelect array

## Abstract

Bread wheat has a large and complex allohexaploid genome with low recombination level at chromosome centromeric and peri-centromeric regions. This significantly hampers ordering of markers, contigs of physical maps and sequence scaffolds and impedes obtaining of high-quality reference genome sequence. Here we report on the construction of high-density and high-resolution radiation hybrid (RH) map of chromosome 4A supported by high-density chromosome deletion map. A total of 119 endosperm-based RH lines of two RH panels and 15 chromosome deletion bin lines were genotyped with 90K iSelect single nucleotide polymorphism (SNP) array. A total of 2316 and 2695 markers were successfully mapped to the 4A RH and deletion maps, respectively. The chromosome deletion map was ordered in 19 bins and allowed precise identification of centromeric region and verification of the RH panel reliability. The 4A-specific RH map comprises 1080 mapping bins and spans 6550.9 cR with a resolution of 0.13 Mb/cR. Significantly higher mapping resolution in the centromeric region was observed as compared to recombination maps. Relatively even distribution of deletion frequency along the chromosome in the RH panel was observed and putative functional centromere was delimited within a region characterized by two SNP markers.

## Introduction

Bread wheat (*Triticum aestivum* L.) is one the four most important crops grown world-wide. The availability of its genome sequence may significantly facilitate breeding for improved yield and resistance to biotic and abiotic stresses to withstand the changing environmental conditions. However, wheat genome sequencing is hampered by its large size (1C ∼ 17 Gb) and allohexaploid nature (AABBDD genome). To facilitate the wheat genome sequencing, the International Wheat Sequencing Consortium (IWGSC^1^) was established in 2005. The main challenge in obtaining a reference sequence of bread wheat is the ability to contiguously order BAC contigs or sequence scaffolds along the chromosomes (Choulet et al., 2014b). To achieve this goal, IWGSC follows a strategy of physical mapping and sequencing of the individual chromosomes and chromosome arms (Eversole et al., 2014). An ideal physical map is fully oriented and anchored to high-resolution genetic map with high marker density (Meyers et al., 2004; Paux et al., 2008a; Ariyadasa and Stein, 2012).

Genetic maps are based on recombination between polymorphic molecular markers. A shortcoming of genetic maps is the variation in recombination rate along chromosomes and, in particular, strong suppression of recombination in peri-centromeric regions (Korol, 2013). About one-third of the wheat genome is located in recombination-poor regions (Akhunov et al., 2003). Thus, the order of loci within centromeric regions cannot be determined merely through recombination mapping. This problem can be solved by using the radiation hybrid (RH) mapping approach (Kumar et al., 2012a).

Radiation hybrid mapping is a physical mapping approach based on radiation-induced deletions for mapping markers (Michalak de Jimenez et al., 2013). A panel of independently derived RHs is assayed for the presence or absence of marker loci, and the patterns and frequencies of marker co-retention are used to calculate their physical proximity and to develop a RH map. RH mapping method has several advantages over genetic mapping: (1) recombination independence, (2) higher resolution in peri-centromeric and centromeric region, and (3) polymorphic marker independence (Kumar et al., 2014). Because of these advantages, RH mapping was used to anchor BAC (Bacterial Artificial Chromosome) contigs on chromosomes 3B and 6B of wheat (Paux et al., 2008b; Kobayashi et al., 2015).

Molecular markers are a critical component needed to anchor and orient physical maps. To anchor a given contig, one marker locus placed on a map is the necessary minimum, although two are required for proper contig orientation. Despite the fact that physical maps have improved over the past few years and the number of contigs covering a chromosome or whole genome has been decreasing (Frenkel et al., 2010), their number may still exceed several hundred per chromosome. Thus, hundreds of evenly distributed markers are needed and, in order to achieve a proper orientation of the contigs, the number of markers must be doubled (Kumar et al., 2012a). A similar situation concerns anchoring, orienting, and eventually merging of sequence scaffolds in genome sequencing projects (e.g., Choulet et al., 2014a). To satisfy this need, high-throughput mapping systems such as array based platforms have been employed (Ariyadasa and Stein, 2012). Currently, the 90K Illumina iSelect chip (Wang et al., 2014) is one of arrays with highest number of available SNP (single nucleotide polymorphism) markers in wheat. The chip allows mapping of approximately 2000 marker loci per chromosome (Wang et al., 2014). In ideal cases, it could allow anchoring and orienting of one thousand physical contigs per chromosome. Additionally, the high density maps with high resolution can facilitate precise mapping and cloning of agronomically important genes. For example, chromosome 4A of wheat has been reported to harbor over 50 genes involved in regulation of grain yield and quality, reaction to biotic and abiotic stresses, and genes regulation of physiological traits such as height, maturity and dormancy (Araki et al., 1999; Börner et al., 2002; McCartney et al., 2005; Jakobson et al., 2006; Shorinola et al., 2016).

In this work, two endosperm radiation hybrid (ERH) panels consisting of 1069 lines were developed for wheat chromosome 4A as well as chromosome 4A deletion map. Selected lines of the ERH panels and wheat chromosome deletion bin lines were genotyped by the 90K iSelect chip (Wang et al., 2014) and corresponding maps were constructed. This work is a part of 4A project to aid development of a complete reference sequence of this chromosome.

## Materials and Methods

### Plant Material

Endosperm radiation hybrid panels were developed from two independent crosses between hexaploid wheat (*T. aestivum*, L.) cultivar ‘Chinese Spring’ (CS) whose pollen was irradiated and nulli-tetrasomic (NT) lines N4AT4B and N4AT4D, which served as female parents (Supplementary Figure S1). Fifteen chromosome deletion lines of CS chromosome 4A (four for short arm and 11 for long arm; Endo and Gill, 1996; **Table 1**) provided by the NBRP-WHEAT Centre (Japan) were used for chromosome deletion bin map construction and validation of RH maps. The 4AS and 4AL chromosome arms were flow sorted from the 4A double ditelosomic line of CS in which chromosome 4A is represented by a pair of telosomes representing the short (4AS) and the long arm (4AL) of 4A (Sears and Sears, 1978). Grains of this stock and CS were kindly provided by Dr. Bikram S. Gill (Department of Plant Pathology, Kansas State University, Manhattan, NY, USA).

**Table 1:** 4A deletion stock with their fragment length and accession number.

Deletion stock	Breakpoint	Accession number
4AS-01	0.20	LPGKU1156
4AS-02	0.71	LPGKU1157
4AS-03	0.76	LPGKU1158
4AS-04	0.63	LPGKU1159
4AL-01	0.85	LPGKU1143
4AL-02	0.75	LPGKU1144
4AL-04	0.80	LPGKU1146
4AL-05	0.66	LPGKU1147
4AL-06	0.84	LPGKU1148
4AL-07	0.66	LPGKU1149
4AL-09	0.73	LPGKU1150
4AL-10	0.82	LPGKU1151
4AL-11	0.66	LPGKU1152
4AL-13	0.59	LPGKU1154
4AL-14	0.79	LPGKU1155

### ERH Panels Development

Endosperm radiation hybrid panels were prepared as described by Kumar et al. (2012a) and Tiwari et al. (2012). Briefly, dehiscent spikes of CS were irradiated using gamma rays (10 Gy, 15 Gy, or 20 Gy) and the pollen from the irradiated spikes was immediately used to pollinate previously emasculated spikes of N4AT4B and N4AT4D lines (Supplementary Figure S1). The irradiation was done using an Acel Gamma Cell 220 irradiator (Gamma Irradiation Facility, North Dakota State University, Fargo, ND, USA). Seeds were harvested 20 days after pollination and endosperm was dissected from each seed as described by Tiwari et al. (2012). Endosperms were individually placed in microtubes and stored at -80°C until DNA extraction.

### DNA Extraction and Characterization of ERH Panels

DNA extraction from plant tissues and endosperm of the 4A RH panels was done using Invisorb^®^ Spin Plant Mini Kit (Stratec Biomedical, Berlin, Germany) following the manufacturer’s protocol. Leaf tissues from chromosome deletion lines and NT stocks were harvested from 3 weeks-old seedlings and lyophilized. Dry tissues were homogenized using two 5 mm glass beads and MM3 mill (Retsch, Haan, Germany). The homogenization was done 4 min and 27 Hz. Similarly, the endosperm tissues of individual 4A RH lines were desiccated and homogenized two times for 4 min at 27 Hz. The amount and quality of extracted DNA samples was checked using a NanoDrop Spectrophotometer (Thermo Scientific, Waltham, MA, USA) and electrophoresis in 0.8% agarose gel, respectively.

Telocentric chromosomes 4AS and 4AL were flow-sorted as described by Kubaláková et al. (2002) and their DNA amplified by multiple displacement amplification as described by Šimková et al. (2008).

Equivalent of 4A monosomic lines in N4AT4B and N4AT4D genetic background were used as controls (**Table 2**). The 4A monosomic lines were reconstructed by equimolar pooling of 10 ERH lines which showed no deletions (100% of marker retention) for each genetic background.

**Table 2:** Controls included in single nucleotide polymorphism (SNP) genotyping.

Control	Number of 4A copies
CS	2
Mono4ATetra4B	1
Mono4ATetra4D	1
N4AT4B	0
N4AT4D	0
4AS	Short arm
4AL	Long arm

### Panel of 4AS- and 4AL-Specific Markers Design and ERH Lines Verification and Characterization

To select ERH lines with maximal informative content, a set of reliable 4A-specific sequence-tagged sites (STS) markers was developed. Chromosomal distribution of the marker candidate loci was deduced from the 4A GenomeZipper (Hernandez et al., 2012). mRNA sequences of rice genes in the selected loci (**Table 3**) were used to annotate homologous wheat genes in the linked Chinese Spring chromosome survey (CSS) sequences scaffolds (IWGSC, 2014) of all three wheat sub-genomes. For each gene, at least two pairs of primers were designed in the most variable regions. Specificity of the primers for 4A was tested on DNA of NT lines and chromosome deletion lines by multiplex PCR with an internal standard *owm37* (F: CAGACACGAGATTTGATAAGGCTA, R:TGCTGAAAACACTCTTTCAACAC). PCR reactions were carried out in 20 μl of reaction volume using 15 ng of genomic DNA, 100 mM Tris-HCl, 500 mM KCl, 10 mM MgCl_2_, 1% Triton X-100), 1 μM primers, 200 μM dNTPs (Invitrogen, Waltham, MA, USA), 0.01% Cresol red (Sigma–Aldrich, Dorset, UK), 1.5% sucrose (Lachner, Neratovice, Czech Republic) and 0.5 units of Taq polymerase (Finzyme, Vantaa, Finland). PCR conditions were used as follow: initial denaturation 95°C for 5 min, 40 cycles of: 30 s at 95°C, 30 s at 60°C, 60 s at 72°C, followed by a final extension at 72°C for 10 min. The PCR products were separated on a 4% non-denaturing polyacrylamide gel and visualized using ethidium bromide staining.

**Table 3:** Panel of chromosome 4A-specific STS markers.

Rice gene	SCC scaffold	Marker	Primer sequence	4A deletion bin
Os03g0180400	4AS_5944160	**owm160**	AAGGGCCCATATCATATCACAC	4AS3 – 0.76-1
LOC_Os03g08280			AACAGTGGAGGGCTTTGCTA	
Os03g0161800	4AS_5934783	**owm161**	TTTTCAAGCAGGTTTTGTGC	4AS3 – 0.76-1
LOC_Os03g06620			TCACTTCTCTTCTTTGCGTTCA	
Os03g0203700	4AS_5934571	**owm129**	TGATGATACCAGAGCGTACAAT	4AS3 – 0.76-1
LOC_Os03g10640			CCTTTGATAAGAGGCCCTCAG	
Os03g0297400	4AS_6015094	**owm126**	CCAGTCAGAAATTATTATGAACCTATC	4AS1 – 0.20–0.63
LOC_Os03g18590			CGCTGTCTCGAGATTGGAGT	
		owm127	CAGCAAATGCATGATTTCACTAAT	
			TTCAGATACAGTTCCTGATCTTGC	
Os03g0736300	4AL_7166434	**owm121**	ATTGCCGTCGCGAACTAGA	4ALC – 0–0.59
LOC_Os03g52630			CGGGACGAGCTTGACGAT	
		owm162	TGTTCAAGGACAGCAAGACG	
			CATTTAGATGCTGTCATATTGCTTG	
Os03g0684700	4AL_7176697	owm165	TGAGTTACAGCCACTCTTGTGC	4AL13 – 0.59–0.66 – 1
LOC_Os03g48030			ACCACCTGCCAAGGTTCCTA	
		**owm166**	TGCTACCATGGTTCAGAATGA	
			AGTTGACGAAGCGGCCTTT	
Os03g0854800	4AL_7142517	**owm119**	ACTTGGGAACATTCAGCTCTT	4AL13 – 0.59–0.66 – 1
LOC_Os03g63770			TTTCTCCTCTGTTGGAACATCA	
Os03g0145800	4AL_7091911	**owm167**	TTTTCTTGGTCAGTATAACCTGTTTTT	4AL2 – 0.75–0.79
LOC_Os03g05260			TGAGCAGAGAAAAATTTCCAAG	

*Set of 4A specific STS markers designed for characterisation of ERH panels with distribution supported by 4A GenomeZipper (Hernandez et al., 2012). Markers in bold were used for ERH lines characterization.*

Confirmed chromosome 4A-specific markers and the internal standard were used for characterization of 414 ERH lines in a multiplex setup. The multiplex PCR reactions were carried out in 10 μl volumes and analyzed as described above. ERH lines with at least one deletion and not missing the whole chromosomal arm were considered for further analysis.

### iSELECT SNP Genotyping and Data Analysis

DNA samples of 119 selected ERH lines and DNA of control lines (**Table 2**) and lines of the chromosome deletion stocks (**Table 1**) were sent for genotyping at USDA-ARS Small Grains Genotyping Center, Fargo^2^. The samples were genotyped with the Illumina iSelect 90K SNP array (Wang et al., 2014), following Akhunov et al. (2009). The control DNA (**Table 2**) and DNA of 15 chromosome deletion lines (**Table 1**), were genotyped in three replicates.

The genotyping results were manually analyzed using Illumina’s GenomeStudio (GS) v2011.1 software. In this case, identification of signal differentiated to four categories in the workspace was expected. The categories were: (1) no signal (deleted marker locus: NT, RH, and cytological deletion lines with absence of the marker locus), (2) signal representing monosomic 4A chromosome (reconstructed 4A monosomic lines, arm-specific DNA and RH lines with presence of marker locus), (3) disomic 4A chromosome (chromosome deletion lines with presence of marker locus and CS), and (4) questionable signals. The module of GS for mapping in tetraploid species which allows discrimination of five different signal clusters was used. Each genotype call was manually evaluated and clustered in one of the above mentioned categories manually selected based on signal intensity. Clustering decisions were made according to the signal level from the controls including the 4A nullisomic, monosomic, and disomic lines (**Table 2**; Supplementary Figure S2). The resulting genotypes were exported to MS Excel sheet and the genotypes were converted to binary codes where questionable signals were labeled as missing data.

### iSELECT 90K SNP Cytological Deletion and RH Maps

#### Verification of Chromosome Deletion Map

**FIGURE 1:**
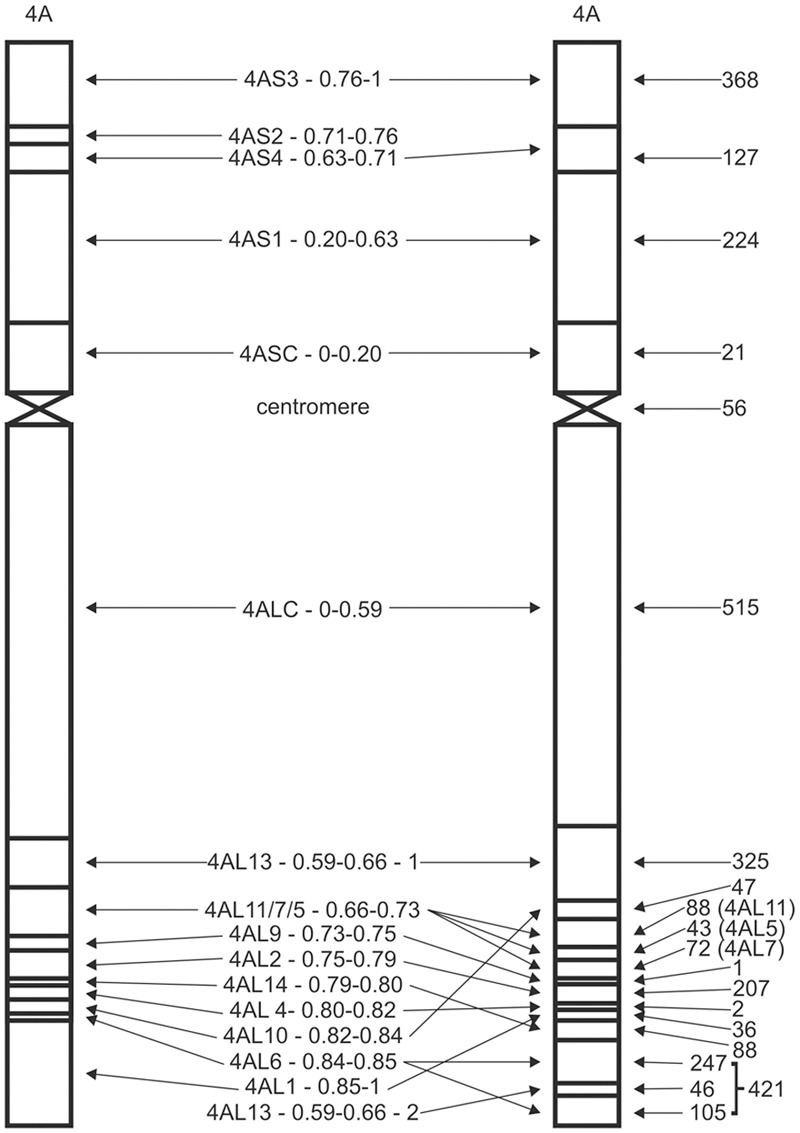
**4A deletion map.** Schematic diagrams of 4A deletion map as developed by Endo and Gill (1996), left, and the deletion map deduced from the 90K iSelect SNP mapping, right. Names of the bins are between deletion maps. The numbers on the right side represent the numbers of assigned SNP markers. Bins sizes are not proportional. Please note several repositions at the distal end of the 4AL arm. The very distal 4AL bin has been split to two by the 4AL13 – 0.59–0.66 – 2 bin and comprises 421 SNP markers out of which 69 could not be ordered with respect to the split bin. A true centromeric bin was defined within 56 markers based on overlap of 4AS and 4AL telosomes.

The CS chromosome deletion lines (**Figure 1**; Endo and Gill, 1996) were genotyped in three replicates. Deletion lines which exhibit disomic-like genotype were included in the CS-like cluster and deletion lines with 4A nullisomic-like signals were designated as members of NT-like cluster (Supplementary Figure S2). Clusters were considered reliable when at least two control replicates were in the cluster. Signals of SNP markers which cannot be clearly assigned to either cluster were marked as questionable and were not included in the map construction.

Assignment of markers to cytological deletion bins was based on an MS Excel ordering tool. Markers specific to a particular chromosome arm were verified by comparing its cluster position with the marker signals on the 4AS and 4AL chromosome specific DNAs. Markers specific for the arm, but missing in all deletion lines, were assigned to the most distal deletion bin of the arm. Additionally, markers common for both arm DNAs and present in all deletion lines were assigned to the centromeric bin defined as an overlap between the 4AS and 4AL telosomic chromosomes. The arm specific peri-centromeric bins were defined as group of markers specific for the chromosomal arm and common for all deletion lines, but not present in the centromeric bin.

#### RH Map

Direct ordering of all markers based on minimizing the map length (calculated as sum of RH distances between sequential markers; Newell et al., 1998) failed due to artificially small distances caused by missing data (pmissing is 0.08, STDV is 0.06) and highly varying marker presence (pA) frequencies (analogous to allele segregation in genetic mapping: average pA is 0.75, STDV is 0.09). Data filtering based on the assumption that co-segregating markers (“twins”) are more reliable for mapping than singleton markers (Ronin et al., 2015) also failed because only a small number of “exact twins” of markers were found in this dataset.

For the RH map construction combined data of B and D panels (2711 markers) were used. Markers with missing genotypes in more than 50 lines and markers having pA higher than 0.8 or smaller than 0.7 were temporally excluded from the analysis. The remaining 1405 markers were clustered by single-linkage algorithm with a cut-off for the proportion of difference in genotypes (at lines where genotypes were not missed in both markers) equals to 0.06.

As chromosome structure is linear, marker clusters with non-linear structure were considered as problematic and subdivided into parts with linear structure. This linearization editing was conducted using LTC software (Frenkel et al., 2010). Markers corresponding to nodes from the diametric paths of the resulted linear clusters were used as candidate skeleton markers. Using MultiPoint program (Mester et al., 2003; Ronin et al., 2010), the set of skeleton markers was globally ordered and further edited to exclude markers causing (i) local map instability upon jackknife re-sampling and (ii) violation of local monotonic increase of distance along the map, and include additional markers that fit well into large or moderate gaps. Coordinates of the obtained 144 skeleton markers were calculated as cumulative sum of distances between adjacent markers. The resulted skeletal map was verified using the bin map.

For the remaining non-skeleton markers, their distances for all skeleton markers were calculated using RH formula (Cox et al., 1990) and Haldane formula for simple recombination event, because at small distances the corresponding two mapping metrics result in highly correlated distances. A marker was attached to the RH map if the Haldane distance to the closest skeleton map marker was less than “10 cM.” Markers, which did not fit this condition, were added to larger map regions using their bin map positions.

The resulting RH map was manually checked taking into the account the position in chromosomal deletion bin map and RH genotype. The STS markers used for ERH line characterization were added. Markers with higher level of missing data and problematic lines were excluded before final map length calculation.

### Mapping of SNP Markers to Chromosome Survey Sequences

To verify marker specificity for chromosome 4A and its regions, comparative analysis was performed with survey sequences from chromosome arms 4AS, 4AL, 4BS, 4BL, 4DS, 4DL, 5AS, 5AL, 5BS, 5BL, 5DS, 5DL, 7AS, 7AL, 7BS, 7BL, 7DS, and 7DL (IWGSC, 2014). The arm sequences were repeat masked using the mips-REdat database (Nussbaumer et al., 2013) and Vmatch software (Abouelhoda et al., 2002). The alignments between marker sequences and the wheat chromosome arm sequences were performed using the BLAST algorithm (Altschul et al., 1990). The BLAST outputs were post-processed by an in-house perl script, which filtered the results based on the following criteria: a minimum identity 90% and a minimum alignment length of 50 base pairs. Finally, the best hit for each alignment was selected.

## Results

### 4AS and 4AL Specific Marker Panel for Characterization of ERH Lines

Selection of RH lines with the highest information content (high number of small deletions, but not missing whole chromosome 4A, or its arm) was ensured by a set of 23 primer pairs that were designed for five and six STS specific for 4AS and 4AL chromosomal arms, respectively. Even distribution of the loci along chromosome 4A was facilitated by 4A GenomeZipper (Hernandez et al., 2012). Five and six primer pairs were found specific for four loci on each 4AS and 4AL chromosome arms, respectively, and denominated as *owm119 – owm167* markers (**Table 3**). Eight most reliable markers, one for each locus were used for characterization of the ERH panels (**Table 3**).

### ERH Panels Development and Characterization

A total of 1069 ERH lines specific for chromosome 4A were developed using three dosages of radiation (10, 15, and 20 Gy; **Table 4**). According to the female parent used (N4AT4B or N4AT4D), the lines were divided into two ERH panels (Supplementary Figure S1). As expected, the recovery of ERH lines negatively correlated with radiation dosage (**Table 4**). DNA was extracted from endosperm of 414 ERH lines representing all three irradiation levels (**Table 4**). A total of 140, 137, 138 lines belonging to 10, 15, and 20 Gy panels, respectively, were characterized with eight 4A-specific STS markers (**Table 3**) using multiplex PCR with internal standard. Estimation of the retention frequency (Kumar et al., 2012a), based on the eight STS markers, showed that 7.1% lines lost the entire chromosome (retention frequency = 0%), whereas 27.1% of the lines did not show any deletion on chromosome 4A (retention frequency = 100%). Additionally, 17.7 and 19.2% of the lines lost complete short and long arm of chromosome 4A, respectively (Supplementary Table S1). A total of 113 lines, 57 and 56 for N4AT4B and N4AT4D ERH panel, respectively, were selected for genotyping by 90K iSelect chip (Wang et al., 2014). The main criterions for selection were the amount of available DNA (at least 2 μg), retention frequency (10–90%) and homogeneous distribution of lines across the three irradiation doses and both female parents. In addition, six lines showing 100% retention frequency were randomly selected from 20 Gy panels (**Table 4**).

**Table 4:** Distribution of endosperm radiation hybrid (ERH) lines across each step of experiment.

	ERH lines prepared		DNA extracted		ERH lines genotyped	
Dose (Gy)	N4AT4B	N4AT4D	N4AT4B	N4AT4D	N4AT4B	N4AT4D
10	250	339	72	68	12	15
15	69	163	68	68	26	24
20	105	143	70	68	22	20
Total	424	645	210	204	60	59

### Marker Calling and RH Map Construction

The selected 119 ERH lines were genotyped along with DNA of three replicates of 15 chromosome deletion lines, five control lines and DNA amplified from flow-sorted 4AS and 4AL arms (**Tables 1** and **2**). The samples were genotyped with the iSelect chip (Wang et al., 2014) and genotypes of 81587 marker loci were retained. Manual analysis was found the most effective, as it allowed resolving clusters with non-standard shape and individually judge questionable signals. Additionally, average distance between clusters, especially for clusters with nullisomic and monosomic signals, changed significantly from sample to sample (Supplementary Figure S2). The analysis yielded 2711 SNP markers specific for CS chromosome 4A and polymorphic for the ERH panels. Six and eight markers were found polymorphic only for the N4AT4B and N4AT4D based ERH lines, respectively. A total of 2697 SNP markers (>99%) were common for both ERH panels. Based on the results of SNP genotyping, a total of 23 ERH lines were excluded from RH map construction. These included ten N4AT4B and eight N4AT4D lines, either with 100% retention frequency, or with questionable calls across all markers.

### Verification Chromosomal Deletion Map

Construction of the 4A deletion map was included in this study to verify marker ordering along the chromosome arms in RH map. First, chromosome 4A-specific SNPs were assigned to corresponding chromosome deletion bins based on their presence in chromosome deletion lines (**Table 1**). Out of 2711 4A-specific markers, 24 were excluded because of unreliable signal calling. In total 2687 SNP markers were assigned to 16 different deletion bins (**Figure 1**; Supplementary Table S2). Surprisingly, no SNP was assigned to 4AS2 – 0.71–0.76 bin and only one SNP was assigned to 4AL9 – 0.73–0.75 bin. The highest number of SNPs (515) was assigned to 4ALC – 0–0.59 bin (**Figure 1**; Supplementary Table S2). The large number of markers offered high resolution and, in combination with DNA obtained from telocentric chromosomes 4AS and 4AL, allowed identification of a new deletion bin in 4AL telomeric region and precise characterization of centromeric bin of the CS 4A chromosome. Besides the new bin identification, the higher resolution enabled better characterization of bins and their reordering (**Figure 1**; Supplementary Table S3). Linear order of 4AS bins was compared against deletion maps published at Graingenes^3^, and confirmed previous findings. On the other hand, the order of bins in the distal part of the 4AL chromosome arm changed significantly. Bins, 4AL1 —0.85–0.86, 4AL14 – 0.79–0.80, and 4AL10 – 0.82–0.84 were rearranged (**Figure 1**) and the bin 4AL – 0.66–0.73 previously delimited by deletion lines 4AL-11/7/5 (LPGKU1152, LPGKU1149, LPGKU1147; **Table 1**) was split to three (**Figure 1**; Supplementary Table S3).

#### The New 4AL Distal Chromosomal Deletion Bin

Data analysis showed that the deletion line 4AL-13 (LPGKU1154; **Table 1**) comprises two fragments of the 4AL chromosome arm. The sub-centromeric region represents original observation of Endo and Gill (1996), but its distal end is combined with a short fragment of 4AL sub-telomeric region; this was not reported previously. This finding was confirmed by the fact that 46 markers identified in this new bin were present only in 4AL-13 deletion line, telocentric chromosome 4AL and control lines with complete chromosome 4A. Additionally, sequences of these markers were found homoeologous to chromosome specific CSS of chromosome arms 4AL, 7AS and 7DS (IWGSC, 2014; Supplementary Table S3).

#### 4AL Centromeric Bin

The centromeric bin was defined as overlap of flow-sorted telocentric chromosomes 4AS and 4AL and determined with 56 SNP marker loci (2% of all mapped markers). The bin was named as 4A-centromere. The original centromeric bin of 4A chromosome delimited by the 4AL-13 and 4AS-01 lines (**Table 1**) was partitioned to three bins and increased the number of detected bins to 18 (**Figure 1**). This partition divided marker loci of the original centromeric bin to 21 for 4ASC – 0–0.20 bin, 56 for 4A-centromere and 515 for 4ALC – 0–0.59 bin (**Figure 1**; Supplementary Table S3).

### Radiation Hybrid Map of Chromosome 4A

The RH map was constructed using all 2687 polymorphic SNPs following Newell et al. (1998) and Ronin et al. (2015). In the first step, markers with missing data genotypes in more than 50 lines and markers with highly varying pA frequencies higher than 0.8 or smaller than 0.7 were temporally excluded from the analysis. The remaining 1405 loci were clustered by single-linkage algorithm. Two clusters were obtained, one with 931 and the second with 434 linked marker loci. The remaining 40 loci were singletons (presumably due to higher level of errors). Both clusters were checked for linear structure (Frenkel et al., 2010). Markers corresponding to nodes from diametric paths of the resulted parts with linear clusters were used as candidate skeleton markers. Using MultiPoint program (Mester et al., 2003; Ronin et al., 2010), the skeleton markers were globally ordered and further edited to exclude problematic markers. A total of 144 skeleton markers were obtained and the resulting map was verified using chromosome deletion bin map (Supplementary Figure S3). Remaining non-skeleton markers were added to RH map based on their chromosome deletion bin position. The resulting RH map comprised 2467 markers, which were then manually checked. The final RH map comprises 2316 markers (2308 SNP, 8 STS; Supplementary Table S3) spanning the length of 6550.9 centi Ray (cR). Considering the molecular size of chromosome 4A of 856 Mb (Šafář et al., 2010), the average resolution of the final RH map is 0.13 Mb/cR.

In the 96 informative ERH lines (lines with deletions), the number of deletion events ranged from 1 to 72 per line, with an average of 11.25 deletions per line. For the 4AS arm, 369 deletions were identified with a maximum 31 and an average 3.84 deletions per line. For the 4AL arm, 711 deletions were retained with a maximum 65 and an average 7.41 deletions per line. Average of 1.16 and 1.32 deletions per Mb for 4AS and 4AL chromosome arms, respectively, were observed considering their respective sizes to be 316 and 540 Mb, (Šafář et al., 2010).

**FIGURE 2:**
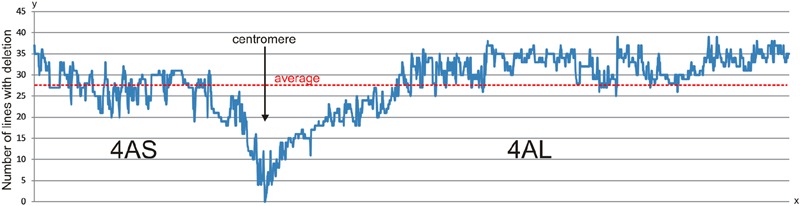
**Deletion rate along the 4A chromosome in 96 RH lines.** The chart shows number of lines with deletion per SNP locus (*y*-axis) along the 4A chromosome based on marker order in the 4A RH map (*x*-axis). The deletion rate is stable in the most of the chromosome arms with significant drop in peri-centromeric regions. The peri-centromeric regions could be delimited as continual decrease below an average line. However, the decrease in the number of identified deletions in the peri-centromeric regions may be also accounted to lower marker density because used markers are associated with transcribed genome regions, only. The zero deletion rate is presumably associated with functional centromere.

The deletion frequency per marker locus in the ERH panels varied from 0 to 39 with an average 27.7 deletions per locus (**Figure 2**). The frequency of deletions along the chromosomal arms was slightly higher for the long arm (average 28.75) compared to short arm (average 25.43) (**Figure 2**). This corresponds with higher deletion rate per line as described above. If we consider regions on the 4A RH map with continuous marker deletion frequency below average and along the centromere as peri-centromeric regions (**Figure 2**), then the 4AS and 4AL peri-centromeric regions comprise 175 and 387 markers, respectively. If we consider, even distribution of the markers along the chromosome, then the 4AL peri-centromeric region would be 2.21-fold longer compared to the 4AS peri-centromeric region (**Figure 2**). This assumption is corroborated by the presence of relevant markers in the centromeric bins of the chromosome deletion map. This would also mean that only portion of 4AS peri-centromeric region flanked by markers *tplb0056l21_172* and *Kukri_c8543_3646* is covered by 4ASC – 0–0.20 deletion bin. The 4AS peri-centromeric region is also included in 4AS1 – 0.20–0.63 and 4AS4 – 0.63–0.71 bins whereas the 4AL peri-centromeric region flanked by markers *Kukri_c42920_338* and *Kukri_rep_c104642_308* is fully covered with the 4ALC – 0–0.59 deletion bin.

### Evaluation of 4A RH and Chromosome Deletion Maps

**FIGURE 3:**
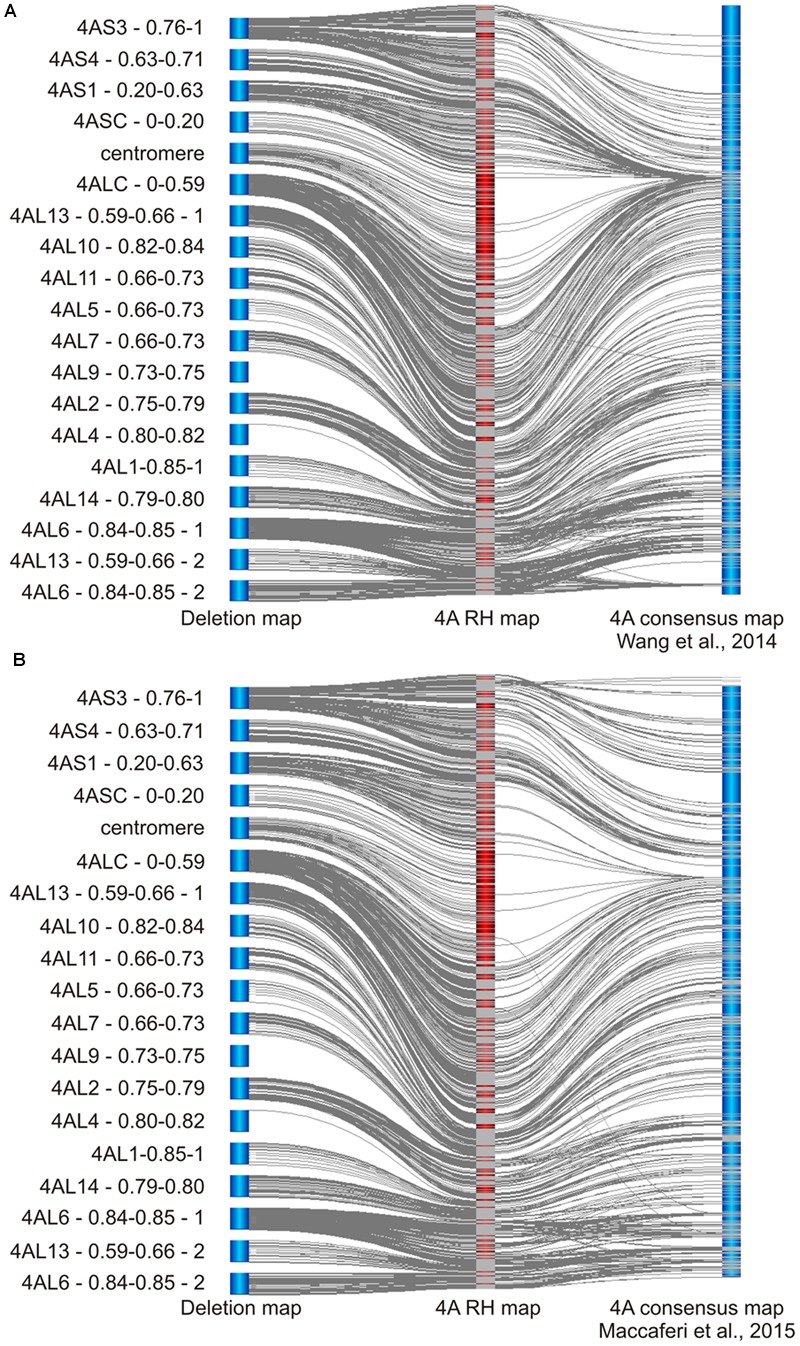
**Comparing of 4A RH map with recombination consensus maps of 4A chromosome of bread and pasta wheats. (A)** Comparison of the deletion map supported 4A RH map with hexaploid bread wheat recombination consensus 4A map (Wang et al., 2014). From the left to right – deletion map, RH map and recombination map. The overall marker order agrees with a few exceptions. The major differences are the significant increase in the resolution and marker density at centromeric region of the RH map, and significant differences at distal ends of the chromosomal arms (e.g., the distal end of the 4AL arm shows inversions and marker translocation). The regions (similarly to the centromere region) show significant decrease in marker synteny exhibited as a lack of lines connecting the common markers. **(B)** Comparison of the deletion map supported 4A RH map with tetraploid durum wheat recombination consensus 4A map (Maccaferri et al., 2015). From the left to right – deletion map, RH map and recombination map. As expected, this map shares fewer markers with the RH map. Most of the features are similar to the comparison shown in **(A)** except for more conserved marker synteny at the end of the 4AL arm. The resolution of this map at the centromeric region seems to be better as compared to the hexaploid recombination map. Gray lines between the maps link positions of the same SNP markers. Centromeres are denominate by the deletion bin “centromere.”

To evaluate the mapping accuracy, resolution, and specificity, we analyzed SNP markers and mapping results using two approaches. First, 4A-specific markers for chromosome deletion and RH maps identified in this study were compared with 4A recombination based consensus genetic maps developed using eight hexaploid (1928 lines) and 13 tetraploid (1332 lines) wheat mapping populations genotyped with the same Illumina platform (**Figure 3**; Wang et al., 2014; Maccaferri et al., 2015). Consensus genetic maps of 4A for hexaploid and tetraploid wheat (Wang et al., 2014; Maccaferri et al., 2015) shared 909 and 677 SNP markers, respectively, with our 4A RH map. In the hexaploid consensus recombination map, markers from deletion bins 4AS4 – 0.63–0.71 to 4ALC – 0–0.59 representing 63.4% of chromosome 4A are mapping to a region of 30 cM representing only 4.6% of the map. On the other hand, in the 4A RH map, the same region represents 3000.3 cR, or 45.8% of the map (**Figure 3A**). In the tetraploid wheat based map the region corresponds to 18.9 cM of the map and represents 10.8% of the map (**Figure 3B**).

Additionally, both recombination maps being in concordance with the RH map suggests that the newly identified 4AL distal bin (based on line 4AL-13; **Table 1**) belongs to the middle part of the most distal 4AL bin denominated by line 4AL-06 (**Figure 3A**; Supplementary Table S3) increasing of number of detected deletion bins to 19 (**Figure 1**). According to the recombination maps, the orientation for a portion of markers from the bins 4AS3 – 0.76-1, 4AL2 – 0.75–0.79, and 4AL6 – 0.84–0.85 - 1 are inverted in the 4A RH map (**Figure 3**). On the other hand, besides the inverted block of markers, there are only few markers (1 and 2 markers in hexaploid and tetraploid consensus map, respectively) misplaced and caused the discrepancy between the recombination consensus maps and the deletion and RH map (**Figure 3**).

The second approach to evaluate the specificity of the mapped SNPs was by BLASTn comparison with survey sequences of individual chromosomes (4B, 4D, 5B, 5D, 7A, and 7D; IWGSC, 2014). A total of 2381 SNP loci could be reliably mapped to chromosome 4A survey sequences. 306 of the SNPs were not homologous with chromosome 4A survey sequences (Supplementary Table S3), but they produced reliable genotyping signals on the 4AS/4AL arm-specific DNA. Also, homology test with homoeologous chromosomes (4B, 4D, 5B, 5D, 7A, and 7D) confirmed the specificity of 132 of the markers to the expected genomic regions (Supplementary Table S3).

## Discussion

Main advantages of RH maps are higher resolution in chromosomal regions with suppressed recombination and a possibility to map markers without a need for polymorphism [reviewed by Kumar et al. (2014)]. In this work, two endosperm-based RH panels were developed as sets of lines in which the irradiated 4A chromosome was in monosomic composition to avoid heterozygosity issues (Supplementary Figure S1). Due to a limited resolution of the 90K SNP iSelect array (Wang et al., 2014) in distinguishing signal levels from chromosomes in nullisomic and monosomic status, a wide range of controls including lines without chromosome 4A (nullisomic), with one copy of 4A (monosomic) and two copies of this chromosome (disomics) were used. Without the controls reliable identification of signal clusters would not be possible in many cases (**Table 2**; Supplementary Figure S2).

### 4A SNP Chromosome Deletion Bin Map

To facilitate and verify marker ordering during RH map construction, 15 deletion lines with defined deletions in chromosome 4A were selected (**Table 1**; Supplementary Table S2) and genotyped with the iSelect chip (Wang et al., 2014). In previous studies 786 EST loci were mapped in 10 4A-specific deletion bins (Miftahudin et al., 2004; Qi et al., 2004). Nine bins were delimited using the deletion lines while the centromeric bin was delimited by markers presence in all deletion lines but absent in NT lines for specific chromosome (Qi et al., 2004).

In the present study, out of the 81587 genotyped SNP markers, 2687 4A-specific SNP markers could be mapped on the 15 deletion lines and 4AL and 4AS telosomes. The mapped markers divided the chromosome 4A into 19 bins (4 and 14 deletion bins for 4AS and 4AL, respectively, and centromere). Reliability of the marker ordering was tested by *in silico* mapping of the markers to homoeologous chromosomal loci (Supplementary Tables S2 and S3). Sixteen bins were identified based on the deletion lines and two new bins were identified because of the chromosomal rearrangement. The centromeric bin was delimited as an overlap between the 4AL and 4AS telosomic chromosomes within the 56 markers (**Figure 1**). In contrast to Qi et al. (2004), this approach allows more precise separation of the centromeric bin from the sub-centromeric bins.

Ordering the deletion map based on chromosomal fraction length (FL) (Endo and Gill, 1996) did not match the SNP marker genotypes. Although this was unexpected observation, such inconsistencies in measurements of chromosomal deletions were reported previously (Qi et al., 2003; Dilbirligi et al., 2004; Sourdille et al., 2004; Philippe et al., 2013; Belova et al., 2014; Akpinar et al., 2015). In this study, a single bin 4AL – 0.66–0.73 previously defined by three lines 4AL-05 (LPGKU1147), 4AL-07 (LPGKU1149), and 4AL-11 (LPGKU1152) was split to three bins containing 43, 72, and 88 SNP loci, respectively (**Figure 1**; Supplementary Table S2). On the other hand, in some lines, even the deleted chromosomal fraction could be distinguished, little or no markers could be mapped in bins defined by them. For example the 4A bins originally defined as 4AS2 – 0.71–0.76, 4AL9 – 0.73–0.75, and 4AL4 – 0.80–0.82 by Endo and Gill (1996) comprised only 0, 1, and 2 SNP markers, respectively (**Figure 1**; **Table 4**). Furthermore, these bins were not supported by any previous cytological deletion mapping studies (Qi et al., 2003, 2004; Miftahudin et al., 2004). These discrepancies could be explained by uneven chromosome condensation resulting in low resolution of cytogenetic measurements during the FL calculations and possibility that the small bin size or DNA content do not allow for suitable SNP discovery according to Wang et al. (2014) approach.

### The New 4AL Distal Bin

A new 4AL distal deletion bin was delimited through identification of telomeric chromosomal fragment attached to the chromosome 4A deletion line 4AL-13 (LPGKU1154; **Figure 1**; Supplementary Table S2). However, construction of the RH map and comparison with the consensus recombination maps indicated that the situation is more complicated and that the region likely originated from the central part of the 4AL6 – 0.84–0.85 bin splitting it into two. This increases the number of 4A deletion bins to 19. Additionally, 45 out of 46 SNPs from this new bin were mapped to survey sequence homoeologs (Supplementary Table S3) in 7AS and 7DS arms, confirming the distal localization of the fragment in the translocated 7BS segment. Endo and Gill (1996) also observed fusion of chromosomal fragments from different chromosomes and such lines were excluded from deletion stocks. However, this seems to be a small fragment that was likely below the detection limit of cytological banding analysis. This finding may explain the observation of Miftahudin et al. (2004) who identified 7AS/7DS homologous segment before 5AL translocated segment of 4AL chromosome arm (Supplementary Figure S4). Identification of the 7AS/7DS segment may be a result of the attachment of the sub-telomeric region in the deletion line 4AL13 (**Figure 1**). This is corroborated by the absence of such 7AS/7DS segment in the RH map. Additionally, one of the ESTs delimiting the region (BE426203) maps to the CSS sequence scaffold 4AL_7170232 which contain SNP marker Tdurum_contig82236_117 mapped to the very distal 4AL bin 4AL6 – 0.84–0.85. Unfortunately, none of other ESTs from the region is homologous with the SNPs or SNP linked CSS sequence scaffolds. These findings will require further verification.

Additional discrepancy with previously published deletion maps represents segment of ancestral 4AS chromosome on the very distal region of present time 4AS chromosome (Supplementary Figure S4, Miftahudin et al., 2004). This segment was not supported by the 4A RH map. Comparison of the ESTs used to identify the region (BE518074 and BE494743) with the CSS sequences (IWGSC, 2014) showed that BE518074 has 100% identity with sequences of all three chromosomes of group 3 and 96% and only 84% identity with the 4AS and 4DS chromosome, respectively. Also the BE494743 EST has only 93 and 89% identity with the 4AS and 4DS chromosomes, respectively. Both ESTs were mapped to 4AS and 4DS arms, only by hybridization^4^. This suggests that the region was most likely assigned incorrectly to the ancestral 4AS because of paralogous sequences. Moreover, the BE518074 EST contains conserved domain PTZ00067 belonging to 40S ribosomal protein S23 subfamily, corroborating a possibility of misleading hybridization signal. However, more evidences will be needed to resolve these inconsistencies.

### Centromeric Bin

Genetic characterization of centromere is difficult since (peri)centromeric regions exhibit low levels of recombination, if any at all (Chen et al., 2008). Several approaches have been used to define centromeric regions in wheat with the deletion bin mapping being the most popular. Most proximal bins of short and long arms are marked as peri-centromeric and centromeric (Qi et al., 2004; Belova et al., 2014; Wang et al., 2015). More precision was acquired by combining deletion bin maps and recombination maps (Marone et al., 2012). In a previous study (Qi et al., 2004), presence of markers in all deletion lines and absence in NT and DT lines led to their assignment to a centromeric bin of the specific arm. In this work, simultaneous presence of markers in the 4AS and 4AL telocentric chromosomes was used to define centromeric region. The centromeric region was delimited by 56 SNP marker loci (Supplementary Table S3). Further narrowing of the region may be possible by using additional ERH lines. As the chromosome requires an intact centromere to survive cell division, markers from the centromere should not be deleted in any of the RH lines. Out of the 56 markers from the centromeric region, only two markers (Excalibur_rep_c94194_201, and Kukri_c51716_802; Supplementary Table S3) were not deleted in the ERH lines (**Figure 2**). The region characterized by these markers could be considered as the region representing functional centromere of chromosome 4A.

### 4A SNP ERH Map

The 4A ERH panel developed in this work comprises 1069 lines obtained after pollinating N4AT4B and N4AT4D lines with irradiated pollen to minimize the risk of bias, if any, due to the presence of either chromosome in tetrasomic condition. The pollen was irradiated at three different dosages to facilitate map construction by implementing lines with different density of breaks. Out of the 414 lines identified by initial screening, 119 lines with the lowest retention frequency were genotyped using 90K iSelect array (Wang et al., 2014). In these lines, the retention frequency of 76% was observed. It was significantly lower when compared to RH panels developed by seed irradiation. In these cases, the average retention frequency was 80–90% (Kumar et al., 2012a,b, 2015; Michalak de Jimenez et al., 2013; Mazaheri et al., 2015). The main reason for higher retention frequencies in seed panels is the fact, that seeds with higher chromosome breakage level would not produce viable plants (Kumar et al., 2014).

The final 4A ERH map comprises 2316 markers with a length of 6550.9 cR. In the ERH panel, a total of 1080 mapping bins were identified. In comparison, the most marker populated wheat genetic maps, at present, developed by the Population Sequencing (POPSEQ) approach and the Axiom Wheat SNP Genotyping Arrays mapped large amount of markers but with limited resolution. The POPSEQ map based on 90 DH lines ordered its 112,687 markers in total of 1335 mapping bins across the whole wheat genome (21 chromosomes), with 71 mapping bins for chromosome 4A (Chapman et al., 2015). The Axiom consensus map ordered 2358 SNPs in only 45 mapping bins of the 4A chromosome (Winfield et al., 2015). This suggests 15 and 24-fold higher resolution power of the 4A ERH map when compared to the POPSEQ and Axiom wheat maps, respectively.

To further validate the 4A ERH map, comparisons with consensus recombination maps of tetraploid and hexaploid wheat developed using the same 90K iSelect SNP array, were made. The consensus recombination maps were constructed using 12 RIL and one DH populations with 1928 lines for tetraploid, and eight DH mapping populations with 1332 lines for hexaploid wheat (Wang et al., 2014; Maccaferri et al., 2015). The 4A consensus map of the hexaploid wheat (Wang et al., 2014) has a length of 652 cM (271 recombination mapping bins) and comprises of 1928 SNP markers from which only 973 mapped to the 4A ERH map. The 4A consensus map of the tetraploid wheat (Maccaferri et al., 2015) has a length of 176.5 cM (320 recombination bins) and contains 1346 SNP markers out of which only 670 mapped to the 4A ERH map. However, none of the recombination mapping populations was derived from cv. Chinese Spring and different levels of marker polymorphism could explain the discrepancy in the number of markers mapped on chromosome 4A.

Because of different mapping populations and map length calculations used in the recombination and RH mapping, a meaningful comparison of the sensitivity and resolution can be done only by comparing the number of mapped markers and number of mapping bins, respectively. The 4A RH map comprises of 1.7- and 1.2-fold more SNP markers as compared to the consensus tetraploid and hexaploid maps, respectively (Wang et al., 2014; Maccaferri et al., 2015). The higher number of mapped markers is due to the advantage of RH mapping, which does not require polymorphism of marker alleles. Another important reason contributing to the higher map resolution was the single chromosome-focused RH mapping approach. The 4A RH map contains 3.4- and 4-fold higher number of mapping bins as compared to the tetraploid and hexaploid recombination maps, respectively. This is despite the fact that our RH map was constructed using 20- and 14-fold fewer lines. Marker order in the recombination maps and the 4A RH map was overall well preserved with only a few exceptions (**Figure 3**). However, the most striking resolution difference was observed in the centromeric and peri-centromeric regions of chromosome 4A (**Figure 3**). This phenomenon was most pronounced in the hexaploid map where the peri-centromeric region in the RH map represents about half of the map, but in the recombination map it is less than 5% of the map (**Figure 3A**). On the other hand, despite the fact that higher and more even frequency of radiation induced deletions along chromosome 4A (**Figure 2**), some interstitial regions of chromosome arms (e.g., region delimited by bin 4AL2 – 0.75–0.79) showed higher resolution in the recombinant maps (**Figure 3A**).

Uneven distribution of recombination frequency along the length of the wheat chromosomes, which usually increases significantly with the growing distance from the centromere were described before (Lukaszewski and Curtis, 1993; Akhunov et al., 2003; Saintenac et al., 2009; Choulet et al., 2014a). The low recombination rate prevents high resolution marker ordering in centromeric regions. The RHs can order markers along the chromosome independently of recombination even in the centromeric regions (Kumar et al., 2014), and our results confirm these findings. The 4A RH map has an even deletion frequency (**Figure 2**) along most of the chromosome 4A, except peri-centromeric regions, where the rate dropped significantly to zero deletions, most likely at the centromere, as described above. Kumar et al. (2015) hypothesized that lower deletion rate in centromeric region may be due to presence of important genes needed for survival. Taking into the account that SNP markers from the 90K iSelect array are largely gene-based (Wang et al., 2014), we can hypothesize that the drop of the deletion frequency in the peri-centromeric regions (**Figure 2**) may be the result of lower gene/marker density and hence detection of fewer deletion events. The low gene density at the centromeric and peri-centromeric regions of the chromosomes has been described previously (Sandhu and Gill, 2002; Qi et al., 2004; Choulet et al., 2010, 2014b). The use of high-throughput markers without sequence preference, such as DArTSeq (Cruz et al., 2013), POPSEQ (Mascher et al., 2013), or ISBP/RJM (Paux et al., 2006), could help clarify these hypotheses.

Additional, discrepancy in marker order between RH and recombination maps was observed in the very distal parts of both chromosome arms (marker translocations and inversions, **Figure 3**). This is in concordance with the higher frequency of recombination in these regions. Different haplotypes of the distal end of the 4AL chromosome arm associated with a loss of alleles and size differentiation of this chromosome in 200 wheat accessions were recently described by Tsõmbalova et al. (2016).

To date, four RH maps have been constructed from single plant chromosomes. However, none of them matches the map constructed in this study in the density and the resolution. There are two RH maps developed for wheat chromosome 3B based on seed panels and either combination of ISBP, SSR and DArT markers on 70 RH lines (Kumar et al., 2012a), or DArT markers on 463 RH lines (Bassi et al., 2013). The first map, with the length of 1871 cR comprises 541 markers. The second map with final length of 2,852 cR was constructed using 696 RH lines and only 140 markers. For comparison, RH map of barley chromosome 3H constructed using 113 EST and RJM markers on 202 RH lines resulted in a map of 3066 cR (Mazaheri et al., 2015). The fourth map was developed for wheat chromosome 2D using 92 RH lines and 25 markers with the final length of 453 cR (Kumar et al., 2012b).

**FIGURE 4:**
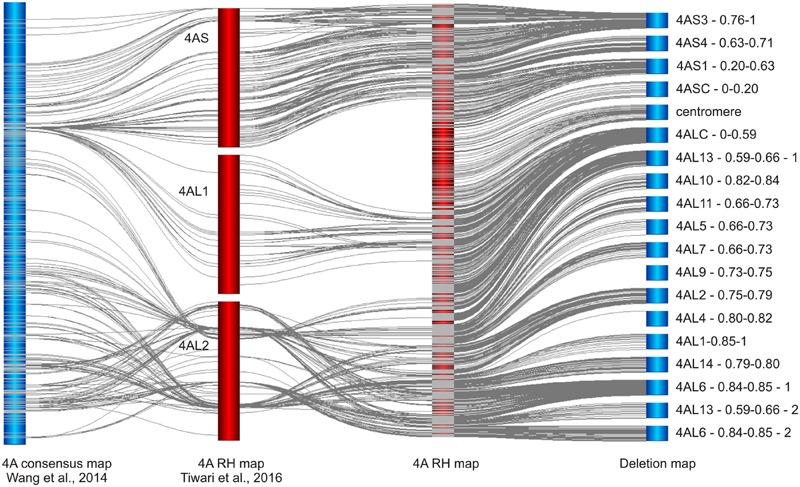
**Comparison of chromosome 4A maps developed in this work with the published 4A SNP maps of bread wheat.** Chromosome 4A RH and deletion maps are compared with the recombination consensus map of 4A chromosome (Wang et al., 2014) and whole genome RH map of bread wheat in which 4A is represented by linkage groups 4AS, 4AL1, and 4AL2 (Tiwari et al., 2016). Note the absence of SNP markers in centromeric region in the whole genome RH map and disagreement in marker order and the number between the whole genome RH map and the remaining maps. All maps were obtained using 90k iSelect SNP array (Wang et al., 2014).

While we have developed RH map of chromosome 4A, Tiwari et al. (2016) developed RH map for the whole wheat genome. Their map was constructed using iSelect 90K SNP array and fertile plants of RH lines derived from hybridization of tetraploid *T. turgidum* cv. Altar 84 and irradiated pollen of cv. Chinese Spring. A total of 833 markers in three linkage groups (one for 4AS and two for 4AL) were identified for chromosome 4A (**Figure 4**) and average mapping resolution was estimated to 2.4 Mb per mapping bin (Tiwari et al., 2016). Out of the 833 markers, 733 mapped in our RH map and 431 in consensus genetic map of 4A developed by Wang et al. (2014). Comparison of the three maps revealed inconsistencies in marker order especially in 4AL2 linkage group (**Figure 4**). Similar to genetic maps, the Tiwari’s RH map comprises small number of markers in centromeric region (**Figure 4**). Markers from our chromosome deletion map mapped in the 4ASC – 0–0.20 bin are not present at all and only 82 markers from the bin 4ALC – 0–0.59 are present and represent the 4AL1 linkage group (**Figure 4**). This shortage limits the utility of the whole genome RH map as a tool for mapping low recombining centromeric regions, at least for chromosome 4A. Additionally, the whole genome approach allowed identification of 35.5% of 4A markers, only. These findings suggest that the whole genome RH mapping in wheat remains challenging and utilization of heterogeneous RH lines may limit reliability and efficiency of marker development and ordering. Our results suggests that the use of reliable high-throughput genotyping platforms with carefully preselected ERH panel and sufficient controls can provide reliable high density maps with significantly higher resolution especially in the centromeric regions.

## Author Contributions

Experimental design: MV, SK, SC, EA, and ABK. Experiments: BB, MŠ, ZF, SC, MA, and AK. Manuscript preparation: BB, MV, ABK, ZF, AK, and JD. Supervision, funding, and reagents: JD, MV, SK, and ABK.

## Conflict of Interest Statement

The authors declare that the research was conducted in the absence of any commercial or financial relationships that could be construed as a potential conflict of interest.

## Footnotes

1 www.wheatgenome.org

2 http://wheat.pw.usda.gov/GenotypingLabs/

3 http://wheat.pw.usda.gov

4 http://wheat.pw.usda.gov
