# Supplementary material for: A High Resolution Radiation Hybrid Map of Wheat Chromosome 4A
Source: Front Plant Sci. 2017 Jan 10;7:2063. doi: 10.3389/fpls.2016.02063 (PMC5222868; doi:10.3389/fpls.2016.02063)
Supplement: Supplementary file 4 [file Image_1.PDF]

Supplementary Material

A High Resolution Radiation Hybrid Map of Wheat Chromosome 4A

Barbora Balcárková[1], Zeev Frenkel[2], Monika Škopová[1, 7], Michael Abrouk[1], Ajay Kumar[3], Shiaoman Chao[4], Shahryar F. Kianian[5], Eduard Akhunov[6], Abraham Korol[2], Jaroslav Doležel[1], Miroslav Valárik[1, *]

***Corresponding author:** Miroslav Valárik, e-mail: valarik@ueb.cas.cz

Supplementary Figures:

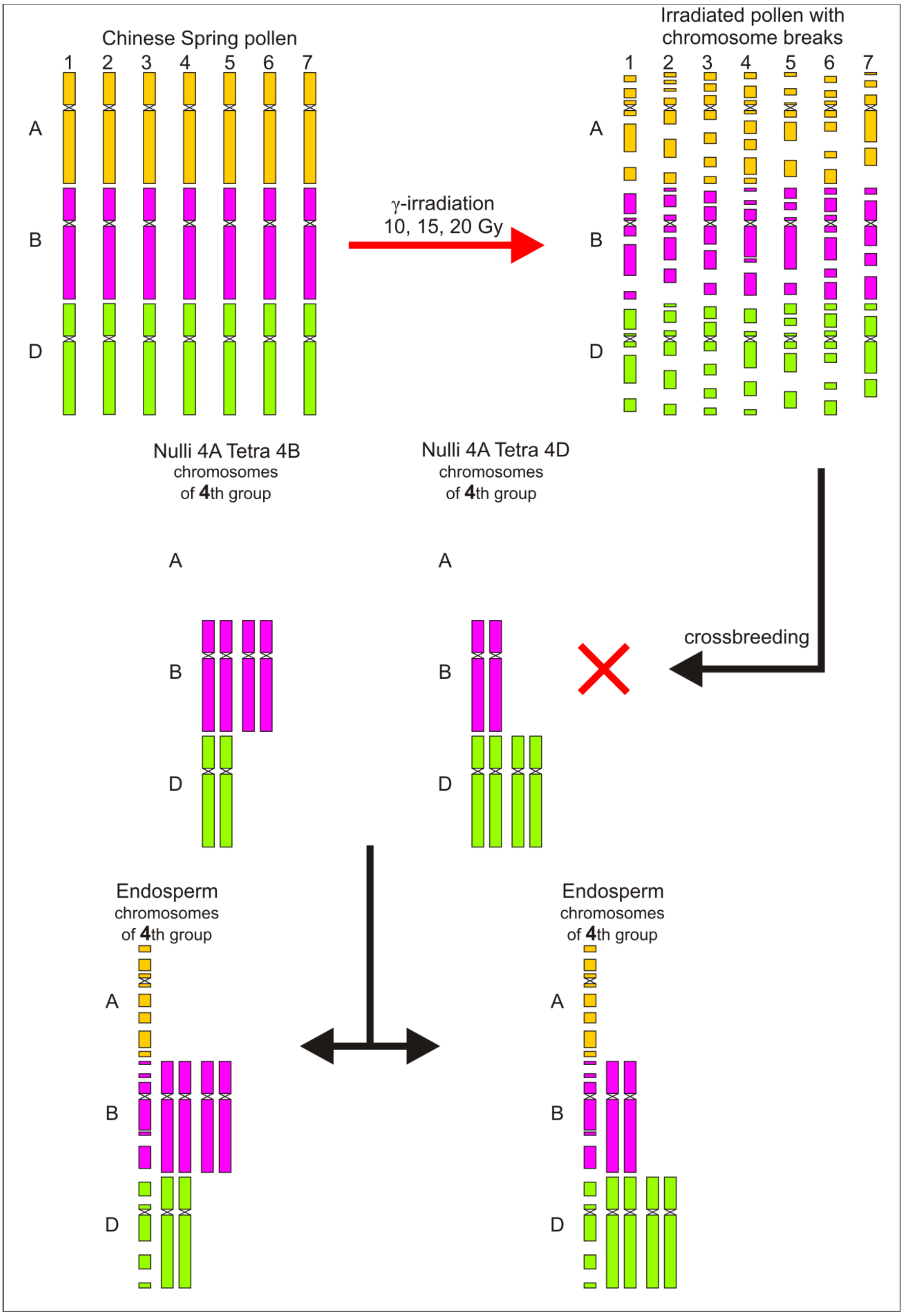

Supplementary Figure S1. Development of the 4A Endosperm Radiation Hybrid panel. Pollen of CS were irradiated and immediately used for pollination of emasculated spikes of N4AT4B and N4AT4D lines to produce two 4A specific Endosperm Radiation Hybrid panels (4ARH).

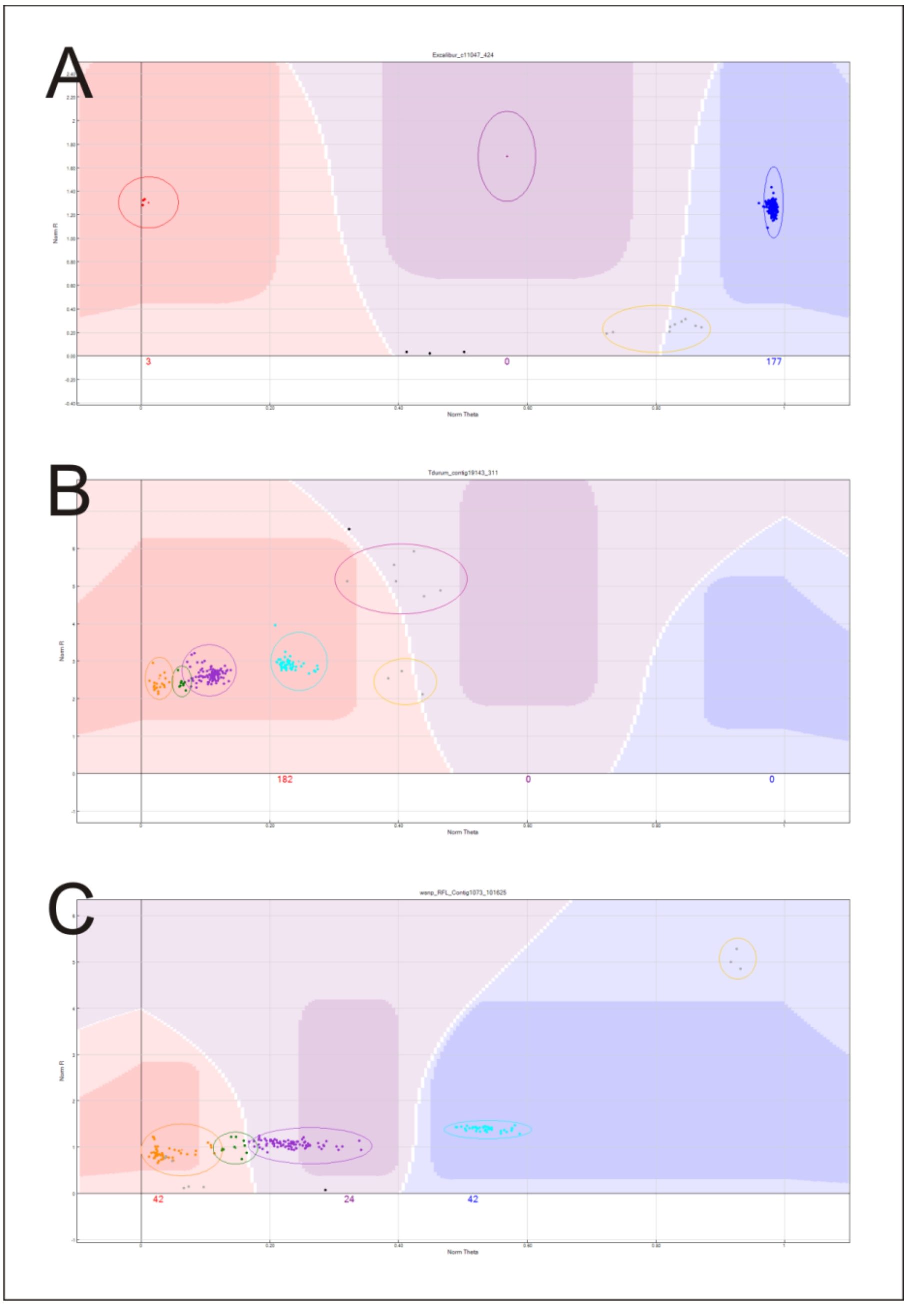

Supplementary Figure S2: Examples of marker clustering (4A specific and non-specific) using the GenomeStudio software.

A module for discriminating of 5 clusters developed for tetraploid species mapping was used for signal clusters delimitation. (**A**) Example of non-specific marker where signals of RH lines cannot be discriminated to individual clusters. (**B, C**) shows examples of 4A specific markers. Signals of the markers from the RH lines and control lines can be separated in distinct clusters. The cyan labelled lines represents CS like signals (disomic 4A). The violet labelled cluster represents composition of genome with monosomic 4A, also confirmed by controls. Cluster of lines with deletion in the tested marker are labelled orange. The cluster was verified by presence of NT lines (no chromosome 4A). Questionable lines were marked green and were excluded from map construction as missing data. The grey dots represent amplified DNA of 4A chromosomal arms.

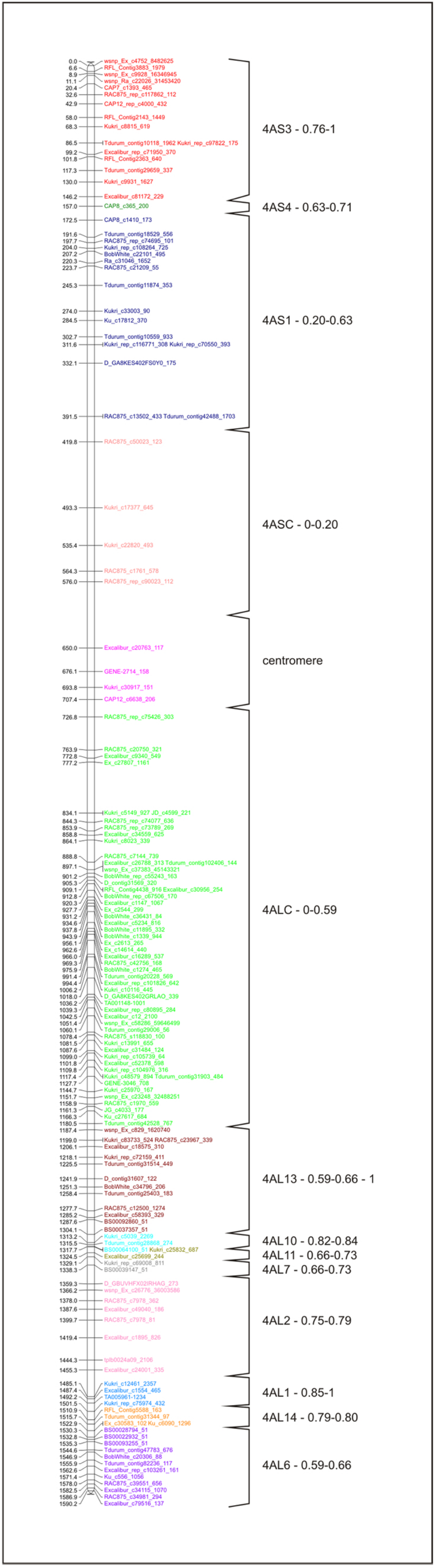

Supplementary Figure S3. Skeleton radiation hybrid map.
The radiation hybrid skeleton map comprising 144 SNP markers spanning 481.25 cRay in length. Markers with different colors belong to different deletion bins. Centromeric markers are in ping color.

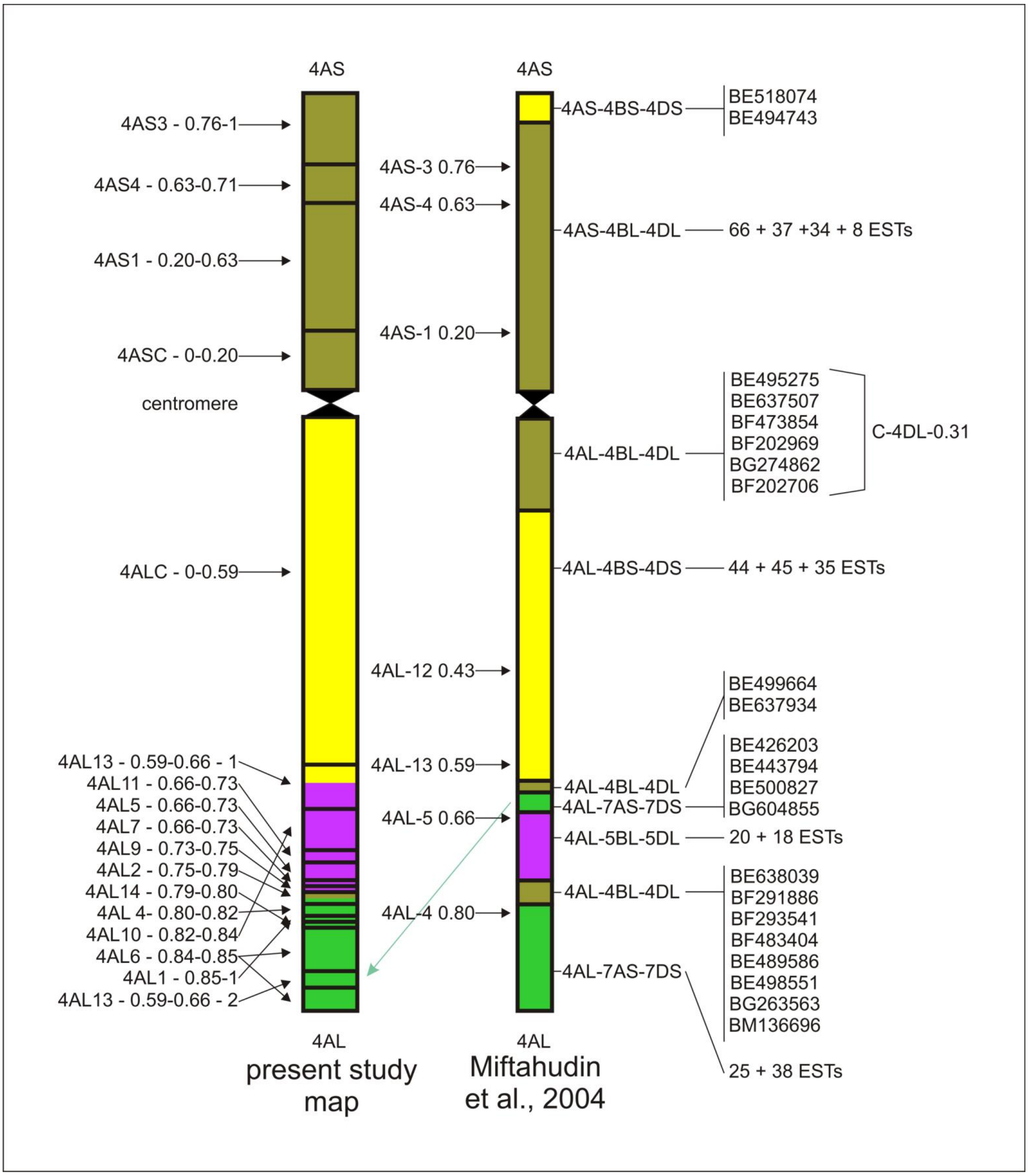

Supplementary Figure S4. Comparison of cytological deletion maps of chromosome 4A. On the left is deletion map derived in the resent study comprising 19 bins with marked bins as inferred from the 90K iSelect array genotyping. The left map is inferred from hybridization of ESTs (Miftahudin et al., 2004) with either EST names or numbers of ESTs supporting the bins (the very right side). Color fill of the chromosomes correspond to large ancestral blocks of chromosome 4A and translocations from 5AL and 7BS chromosomes: 4AS - yellow, 4AL - khaki, 5AL – purple, 7BS - green, and centromere – blue. In the Miftahudin's map the 7BS originating translocated segment is divided to two, but based on the SNPs mapping the proximal 7BS region belongs to the very distal part of the 7BS segment (green arrow). The segment was incorrectly reshuffled because of attachment of distal chromosomal segment to 4A chromosome in the deletion line 4AL13. Also, the ancestral 4AS region at the very distal part of the present time 4AS identified by Miftahudin et al. (2004) was not supported by the SNP mapping.
